# Attosecond Coherence Time Characterization in Hard X-Ray Free-Electron Laser

**DOI:** 10.1038/s41598-020-60328-4

**Published:** 2020-04-06

**Authors:** Guanqun Zhou, Franz-Josef Decker, Yuantao Ding, Yi Jiao, Alberto A. Lutman, Timothy J. Maxwell, Tor O. Raubenheimer, Jiuqing Wang, Aaron J. Holman, Cheng-Ying Tsai, Jerome Y. Wu, Weiwei Wu, Chuan Yang, Moohyun Yoon, Juhao Wu

**Affiliations:** 1 Key Laboratory of Particle Acceleration Physics and Technology, Institute of High Energy Physics, Chinese Academy of Sciences, Beijing, 100049 China; 2 SLAC National Accelerator Laboratory, Stanford University, Stanford, CA 94309 USA; 3 University of Chinese Academy of Sciences, Beijing, 100049 China; 4 Department of Physics and The Pritzker School of Molecular Engineering, University of Chicago, Chicago, IL 60637 USA; 5 Huazhong University of Science and Technology, Wuhan, 430074 China; 6 Jane Lathrop Stanford Middle School, 480 E Meadow Dr, Palo Alto, CA 94306 USA; 7 JSerra Catholic High School, 26351 Junipero Serra Road, San Juan Capistrano, CA 92675 USA; 8 National Synchrotron Radiation Laboratory, University of Science and Technology of China, Hefei, 230029 China; 9 Pohang University of Science and Technology, Pohang, 37673 Korea

**Keywords:** Free-electron lasers, Ultrafast photonics, X-rays

## Abstract

One of the key challenges in scientific researches based on free-electron lasers (FELs) is the characterization of the coherence time of the ultra-fast hard x-ray pulse, which fundamentally influences the interaction process between x-rays and materials. Conventional optical methods, based on autocorrelation, are very difficult to realize due to the lack of mirrors. Here, we experimentally demonstrate a novel method which yields a coherence time of 174.7 attoseconds for the 6.92 keV FEL pulses at the Linac Coherent Light Source. In our experiment, a phase shifter is adopted to control the cross-correlation between x-ray and microbunched electrons. This approach provides critical diagnostics for the temporal coherence of x-ray FELs and is universal for general machine parameters; applicable for wide range of photon energy, radiation brightness, repetition rate and FEL pulse duration.

## Introduction

Femtosecond, high brightness, hard x-ray pulses generated by free-electron lasers (FELs)^1–5^ opens the door to a new frontier of high-intensity x-ray experiments in various research fields, e.g., physics, chemistry^6^, life^7^ and material sciences^8^. These pulses are able to encode valuable structural and dynamical information on atomic scales. Most of the hard x-ray FELs rely on the self-amplified spontaneous emission (SASE) scheme. Due to starting from electron shot-noise, SASE FELs usually provide partially temporal coherence corresponding to temporal isolated spikes^9^. Foreknown information about x-ray coherence time would benefit experiments focusing on ionization dynamics^10^, spectro-holography^11^, nonlinear mixing-wave experiment^12^, etc. Hence, a direct characterization method of the coherence time of the ultra-fast hard x-ray FEL pulse in time domain is a primary requirement in this field.

To directly measure the FEL coherence time, one straight forward way is to implement conventional optical method: autocorrelation, which is a widely used method in the optical wavelength. Typically, the radiation pulse is split into two identical pulses, one pulse is delayed by some optical mirrors and eventually the two pulses are recombined. The output interference versus delay gives the information of coherence time. Obviously, autocorrelation depends on the mirrors to generate optical delays, which limits its applicability for different photon energy ranges. Although it has been proven that a combination of laser beam splitter and mirror based optical delay can be used to implement autocorrelation to characterize the FEL coherence time in the extreme ultraviolet and soft x-ray regime^13,14^; for the hard x-ray pulses, due to the lack of mirrors, it is very difficult to realize autocorrelation. Experiments employing crystals as mirrors to generate an effective delay has been carried out to measure the coherence time of a monochromatized hard x-ray pulse^15^. However, this method cannot be implemented to SASE FEL coherence time characterization, since these crystals lead to external strong purification on the incident pulse spectrum, due to Bragg diffraction. Therefore the measured coherence time will be much longer than the intrinsic SASE FEL coherence time. Hence, currently, there is no effective method to characterize the coherence time of SASE FEL in the time domain. Meanwhile, x-ray pulse duration characterization method based on FEL dynamics has been well established^16,17^, in which the cross-correlation between ‘fresh’ electrons and x-rays has been used to measure the x-ray pulse length. However, since the electrons are almost ‘fresh’, the coherence information is smeared out. Inspired by this idea, we propose the method utilizing the cross-correlation between the x-rays and the microbunched electrons to characterize the coherence time of hard x-ray FEL pulses.

**Figure 1:**
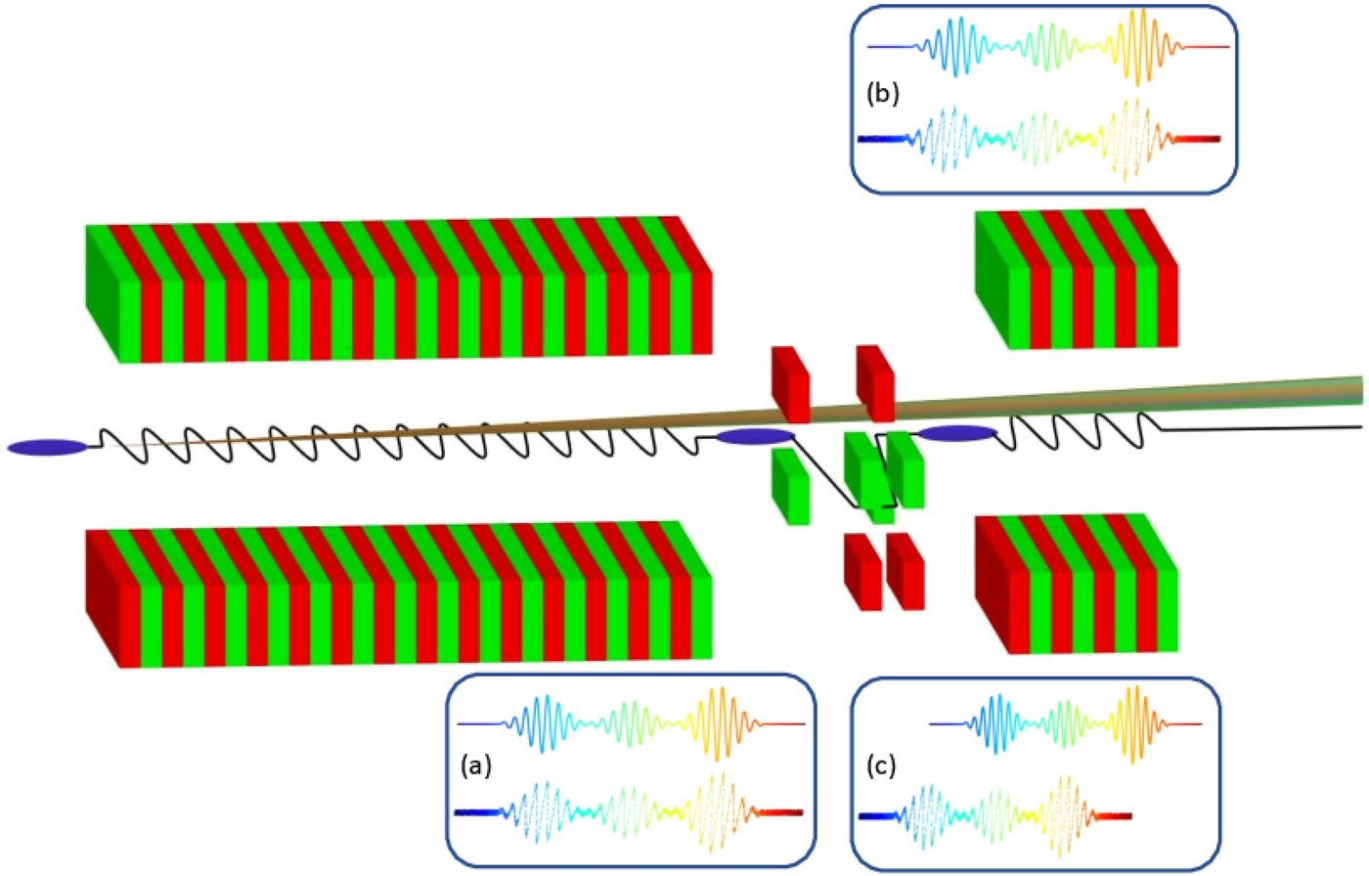
Schematic description of hard x-ray SASE FEL coherence time measurement based on cross-correlation between microbunched electrons and x-ray pulse. Hard x-ray SASE FEL is generated in the before-delay undulators, then a phase shifter is employed to generate a relative delay between the electrons and the x-ray and the final undulator converts the correlation to x-ray pulse energy. (**a**) Visualizing the radiation wave and the microbunched electrons at the beginning of the phase shifter. (**b**) Illustrating the case that the phase shifter induced delay is small. (**c**) Showing that when the delay is quite large, the microbunched electrons would work with x-rays in other coherence spikes.

In this work, we report the first direct measurement result of the coherence time of ultra-fast hard x-ray FEL pulses through a conceptually different approach. In this method, the temporal coherence characteristics of the x-ray pulses are mapped to the cross-correlation between the microbunched electron beam and the x-ray pulse and then the corresponding coherence time can be obtained by decoding the information from the measured cross-correlation. Figure 1 depicts how this coherence time characterization method works, where the whole undulator system is considered as two parts, the first part is where the SASE FEL is generated. The coherence time of the SASE FEL pulse generated from the first part of the undulator is then to be measured in the second part, which converts the correlation information to the x-ray pulse energy. The phase shifter between these two parts is employed to manage the delay between the x-ray pulse and the microbunched electrons to produce and control the correlation.

## Principle

In the following, the essence of this method is described briefly and the details of the theoretical analysis are presented in Sec. Method. In the SASE FEL, a bunch of highly relativistic electrons enters a periodically varying magnetic field, known as undulator, in which the external magnetic field forces the electrons to wiggle transversely and emit spontaneous radiation. This process produces ultrashort bursts of x-rays at the wavelengths ${\lambda }_{r}=\frac{{\lambda }_{u}}{2{\gamma }^{2}}(1+{a}_{u}^{2})$, where *λ*_*u*_ is the undulator period, *γ* is the electron beam Lorentz factor and *a*_*u*_ is the RMS undulator parameter characterizing the undulator strength.

Due to the on-axis speed difference between the electrons and x-rays, the x-ray pulse propagates over the electron beam by one radiation wavelength after one undulator period. While the electron beam traveling through the undulator, the slippage between the x-rays and electrons increases continuously. This increasing slippage *l*_*s*_ determines the cooperative-interaction range between the electrons and the x-rays, and results in coherence spike development. Hence, within the coherence spike, the x-ray and electrons would have a strong correlation^18^. Since these coherent spikes are all evolved from shot noise, x-rays and electrons in different coherence spikes are independent of each other. Now, it is clear that the coherence time of the FEL pulse is determined by the length of the temporal coherence spike. In spite of some coherence enhanced schemes^19–21^, the length of the coherent spike is usually much shorter than the pulse duration for hard x-ray FELs. For example, LCLS, operated in the mode of hard x-ray SASE FEL, generates x-ray pulses with tens of coherence spikes^22–24^, *i.e*., the coherent time is only a few hundredth of x-ray SASE FEL pulse duration.

Based on the above idea, we can trace the correlation between the microbunched electrons and the x-rays by employing a phase shifter to control the delay between them. Then, one more undulator section after the phase shifter, about one FEL gain length, is used to convert the correlation to x-ray pulse energy. It can be expected that, the pulse energy should oscillate corresponding to the delay, but with a decaying oscillation amplitude. The pulse energy oscillation comes from the phase mismatch between the microbunched electrons and x-rays in the scale of x-ray wavelength, similar to two-wave interference model; while the decaying amplitude comes from the decreasing correlation, in the scale of coherence time. This causes that after some point of delay (correlation vanishing point), the oscillation amplitude would become tiny. This characteristic of SASE FEL can be used to experimentally determine the coherence time of hard-xray pulses.

## Results

We carry out experiments to measure the coherence time of hard x-ray pulses at Linac Coherent Light Source (LCLS) with the method we propose above. The experimental setup is based on the normal LCLS SASE FEL configuration^25^. The hard x-ray self-seeding chicane, formed by four dipoles, is employed as the phase shifter. The gas detector is used to record the x-ray pulse energy^26^. By distorting the electron beam orbit with quadruple magnets, we can control the after-delay FEL interaction length to maximize the interference contrast. Hysteresis-loop of magnets has also been carefully considered, so that we only monotonically increase the driving current of the magnetic chicane during the experiment and whenever we need to decrease the current, we do degauss and restart the delay scan from zero. Due to the accuracy of the driving current for the magnetic field of chicane, the step size of the delay scan is set to 0.875 attosecond and the total scan range is set to 525 attoseconds according to the coherence time estimation based on analytical studies^27^. To avoid the stochastic effects in the experiment as much as possible, we record 25 shots of FEL pulses for each delay and use the average value of effective shots (mostly 24 or 25 shots) to represent the pulse energy for the corresponding delay. The photon energy is tuned to be 6.92 keV, the corresponding electron beam energy is 12.48 GeV and other machine parameters are shown in Table 1.

**Table 1:** Key parameters for hard x-ray FEL pulse coherence time characterization.

Parameter	Symbol	Value [Unit]
Beam energy	*γ*_0_*m*_0_*c*^2^	12.48 GeV
Rel. RMS energy spread	*σ*_*δ*_	1.04 × 10^−4^
Norm. transv. emittance	*γ*_0_*ϵ*_*x*,*y*_	0.4*μm* − *rad*
Peak current	*I*_0_	3500 A
Undulator period	*λ*_*u*_	3 cm
Undulator RMS parameter	*a*_*u*_	2.4749
Photon Energy	*E*_*ph*_	6.92 keV

**Figure 2:**
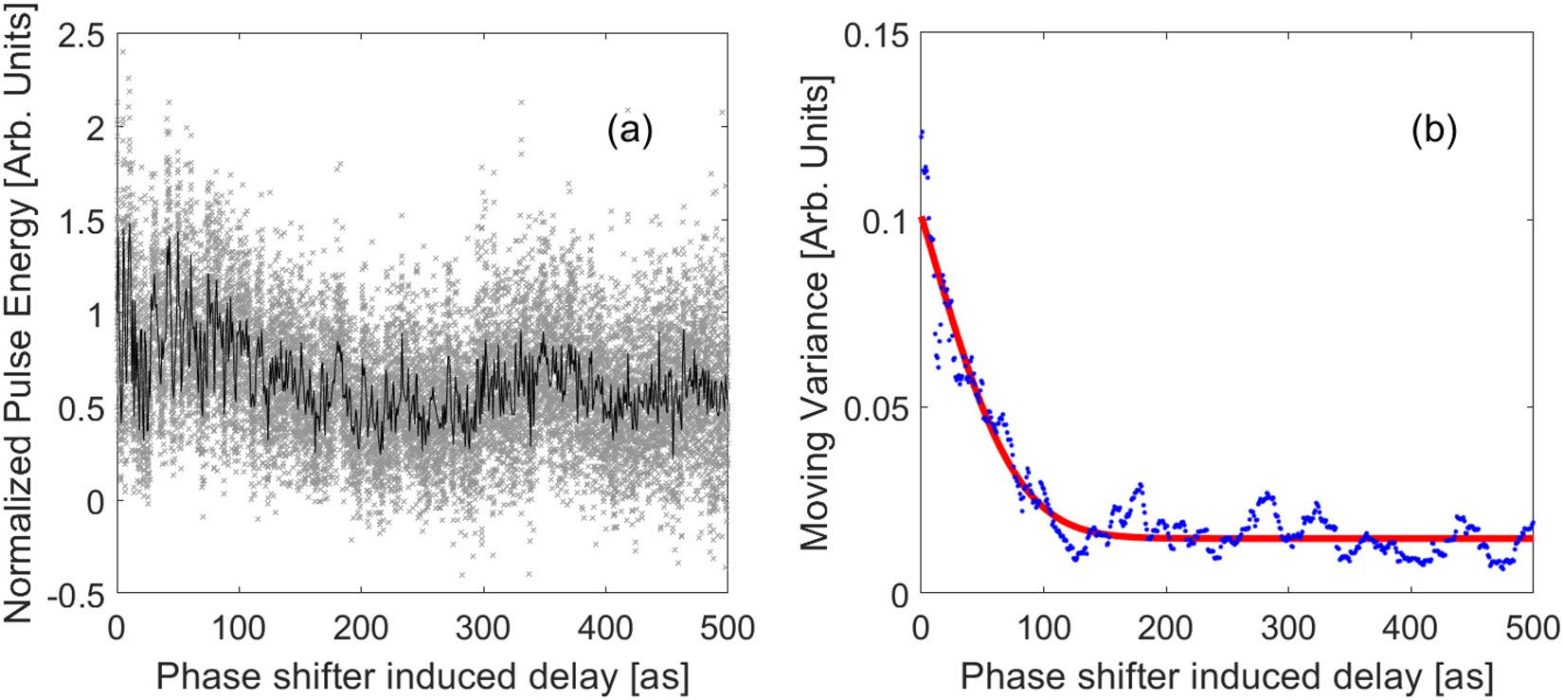
Experimental results of hard x-ray SASE FEL coherence time measurement at LCLS: (**a**) Pulse energy versus delay, in which the black line is the average pulse energy for each delay and the grey points represent the single-shot pulse energy. (**b**) Moving variance with respect to delay, where blue points are obtained by Eq. (6) in Method and the red line is the fitting curve.

In our experiment, total of 15000 shots of FEL pulses are recorded. To get the effective information from the experimental data, first we synchronize the recorded data according to the time stamp and effective shots can be selected by kicking off the outliers according to the measured electron beam charge, orbit and pulse energy. Overall, 0.24% of the recorded shots are invalid and the average pulse energy of these effective shots is about 78 *μ*J. Raw data of the measured pulse energy and number of invalid shots for each delay are shown in Fig. 1 and Fig. 2 in the Supplementary Information, respectively. The measured pulse energy versus delay is shown in Fig. 2(a), in which the black curve is the average pulse energy over effective shots and the grey points are the pulse energy for each shot. According to Eq. (6) in Method, we employ moving variance to extract the oscillation amplitude from the recorded pulse energy and use Gaussian-like function ($Aexp({(t-{t}_{0})}^{2}/2{\sigma }_{t}^{2})+C$) to fit the data to obtain the value of *σ*_*t*_, in which *C* is used to get rid of background noise. The blue line in Fig. 2(b) shows the decaying oscillation amplitude and the red line is the Gaussian-like fitting curve. According to the fitting results, the *σ*_*t*_ is 49.05 attoseconds and the corresponding coherence time is 174.7 attoseconds (see Method, Eq. (7)).

**Figure 3:**
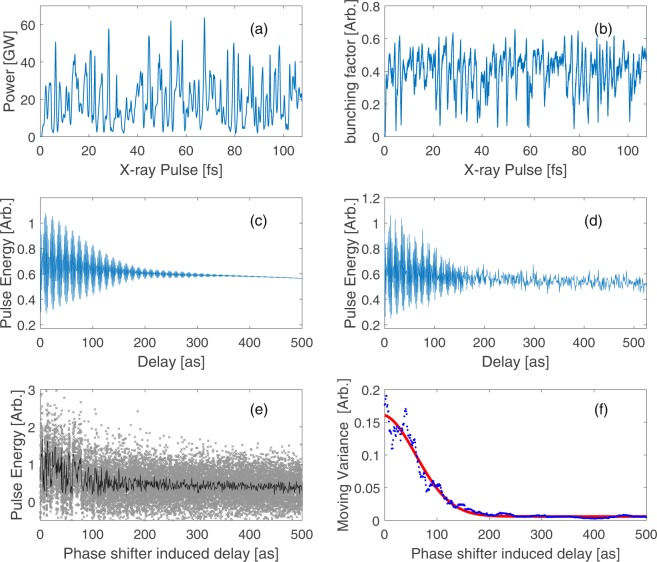
Fluctuation source analysis. (**a**) Time-domain description of hard x-ray SASE FEL pulse. (**b**) Bunching factor profile. (**c**) Pulse energy versus delay (ideal). (**d**) Pulse energy versus delay (initial shot noise fluctuation considered). (**e**) Pulse energy versus delay (experimental fluctuation sources considered), in which the black line is the average pulse energy and the grey points represent the pulse energy for each single-shot. (**f**) The moving variance obtained by Eq. (6) together with its fitting curve.

In Fig. 2(a), the fine pulse energy oscillation we expect is blurred. To find the possible causations, we first simulate the FEL process ideally and then add hypothetical jitters (based on operation experience and physics interpretation) into the system to see whether these jitters would blur the results. We enhance the GENESIS 1.3, a well-benchmarked 3D time-dependent FEL simulation code^28^, to efficiently simulate the FEL process with both phase space stretch and time-delay effects included. The simulation configuration is the same as that of the experiment we mentioned above, which is the baseline of LCLS. Similar to the experiment, we scan the delay from 0 attosecond to 525 attoseconds with a step size of 0.875 attosecond. The temporal profile and bunching factor profile of the zero-delay radiation are shown in Fig. 3(a,b) respectively. Figure 3(a) shows that in the time domain, there are many isolated coherence spikes within a SASE FEL pulse. Figure 3(b) illustrates that the bunching has a similar pattern with the radiation profile. Figure 3(c) shows that if the initial shot-noise is fixed, the fine pulse energy oscillation can be clearly observed. Then, we count shot to shot initial noise fluctuation in and the average of 25 shots is considered as the pulse energy at each delay. As shown in Fig. 3(d), we can find that that if the delay is quite small the pulse energy oscillation mainly comes from the varying correlation between the microbunched electrons and the x-rays; and if the delay is sufficiently long, the pulse energy fluctuation is dominated by shot-noise and the fine pulse energy oscillation disappears.

However, besides the shot to shot initial noise fluctuation, there are a bunch of other experimental fluctuation sources, e.g., spontaneous radiation, electron beam central energy jitter and chicane’s driving current jitter. Based on our experiment result, we estimate the average (detected) spontaneous radiation energy to be about 60 *μ*J and assume it is proportional to the number of electrons we detected for each shot. According to previous operation experience, the relative central energy fluctuation of LCLS electron beam is about 3 × 10^−4^. This central energy fluctuation would be converted to delay fluctuation by the chicane. When the delay is larger than 200 attoseconds, the delay fluctuation will be the same magnitude of wavelength. Besides the electron beam central energy jitter, the chicane’s driving current jitter is another source of delay fluctuation, which is about 1.8 *μ*A empirically. Different from electron beam central energy jitter, the chicane’s driving current jitter can directly affect the delay and this effect can be evident even when the delay is small. Although the delay fluctuation looks small, it has a strong impact on the pulse energy we measured, since the delay jitter affects the interference phase a lot. Compared with normal FEL operations, the chicane’s driving current jitter and electron beam central energy jitter are the main sources of the additional statistical variation. Then, we can try to mimic the experimental situation by adding these fluctuation sources into the ideal simulations mentioned above. The result is shown in Fig. 3(e), in which light gray points represent simulations with different noise at certain delays and the black line is the average radiation energy over different shot-noise cases. It can be found that similar to Fig. 2(a), the fine pulse energy oscillation becomes blurry. With the same data post-process approach base on Eq. (7) in Sec. Method, we can obtain a coherence time of 195.7 attoseconds and the moving variance and Gaussian fitting are shown in Fig. 3(f). Considering the stochastic nature of these fluctuation sources, we randomly add these effects for 10000 times and statistically the coherence time we can obtain is 200.3 ± 36 attoseconds. Although those fluctuation sources blur the fine pulse oscillation, the boundary of interference induced oscillation and shot-noise oscillation, which determines the coherence time, does not change much. The coherence time extracted from the experiment data is still reliable but with a statistical error at magnitude of tens of attoseconds.

## Discussion

For the first time, we directly in time domain measure the coherence time of ultra-fast hard x-ray SASE FEL pulses with a conceptually new approach which we propose and describe in this paper. The method itself fills the vacancy of coherence time characterization in time domain for hard x-ray FELs. Looking forward, the pre-knowledge of coherence time of the x-ray pulse would potentially benefit experiments working on ultra-fast dynamics and structure imaging. It is worthwhile to point out that the approach introduced here is also applicable to seeded FEL, either external seeded^29^ or self-seeding^30^. In that case, it is presumed that a seeded FEL will have fully temporal coherence; yet, due to SASE components, or electron bunch imperfectness, the seeded FEL temporal coherence can be degraded^31^. The approach introduced in this manuscript can then be adopted to check this situation. It should be noted that the measured coherence time is an average result of thousands of FEL shots. It shows the overall longitudinal property of radiation beam based on the current FEL setup rather than a single shot. The method itself is proposed and developed based on FEL physics, which means that it can be applied to any high-gain FELs, like high repetition facilities^32^ and ultra-fast FEL^33^.

## Method

This idea can be described with the well-known one-dimensional model, in which universally scaled collective variables are introduced to describe the FEL dynamics^22^. The normalized radiation field is defined as $A=E/{(4\pi {m}_{e}{c}^{2}\gamma \rho {n}_{e})}^{1/2}$ where *E* is the radiation field, *m*_*e*_ is the electron mass, *c* is the speed of light, *n*_*e*_ is the electron beam density and $\rho ={({a}_{u}{\omega }_{p}/4c{k}_{u})}^{2/3}/\gamma$ is the Pierce parameter, with ${\omega }_{p}={(4\pi {e}^{2}{n}_{e}/{m}_{e})}^{1/2}$, the plasma frequency and *k*_*u*_ = 2*π*∕*λ*_*u*_, the undulator wave number. The bunching factor can be defined as $B=\frac{1}{{N}_{\lambda }}{\sum }_{j=1}^{{N}_{\lambda }}{e}^{-i{\theta }_{j}}$, where *θ*_*j*_ = (*k*_*u*_ + *k*_*r*_)*z* − *ck*_*r*_*t*_*j*_ is the ponderomotive phase, *k*_*r*_ is the radiation wave number, *z* is the coordinate along the undulator axis, *t*_*j*_ is time and *N*_*λ*_ is the number of electrons within one radiation wavelength *λ*_*r*_. As for the dynamics before the phase shifter, it is a typical high-gain SASE FEL process. Since we mainly care about the phase relation between the radiation and the microbunching in the time domain, the steady state model in the following is good enough to describe the FEL dynamics,

1 $$\begin{array}{ccc}\frac{\partial A(\bar{z},t)}{\partial \bar{z}} & = & B(\bar{z},t)\\ \frac{{\partial }^{2}B(\bar{z},t)}{\partial {\bar{z}}^{2}} & = & iA(\bar{z},t)\end{array}$$

where the normalized coordinate variables is introduced, $\bar{z}=z/{l}_{g}$, with *l*_*g*_ = *λ*_*u*_/4*πρ*, the gain length. Since the SASE FEL starts from shot noise, only the noisy bunching factor is considered as the initial condition, which means only *B*(0, *t*) ≠ 0.

For the dynamics in the phase shifter, we only consider the delay effect rather than the phase space stretch induced by the *R*_56_ of the phase shifter, which is quite small compared to the radiation wavelength due to small electron beam energy modulation amplitude. If we denote the radiation field and bunching factor at the end of the undulator, also the phase shifter entrance, as ${A}_{s}({\bar{z}}_{p},t)$ and ${B}_{s}({\bar{z}}_{p},t)$, where ${\bar{z}}_{p}$ is the position of phase shifter; the radiation field and bunching factor at the end of the phase shifter would be ${A}_{s}({\bar{z}}_{p},t)$ and ${B}_{s}({\bar{z}}_{p},t+\Delta t)$ respectively, where Δ*t* is the delay introduced by the phase shifter. Here, we suppose the phase shifter length is negligible. After the phase shifter, we use a short radiator to convert the correlation between the microbunched electron beam and the x-ray to pulse energy. We can regard the process in the after-delay part as coherent emission, since the radiator length is set to be around one gain length only to maximize the interference contrast. The derivation for this choice is given below in Sec. Method. During the coherent emission, the electron beam bunching factor can be regarded as a constant and the radiation field keeps increasing. Namely, only the first equation in Eq. (1) is valid with the bunching term as a constant. In this way, we get the radiation field ${A}_{c}(\bar{z},t)$ at the end of the radiator as,

2 $${A}_{c}(\bar{z},t)={A}_{s}({\bar{z}}_{p},t)+{B}_{s}({\bar{z}}_{p},t+\Delta t)(\bar{z}-{\bar{z}}_{p})$$

It is a good approximation that the SASE radiation field at entrance of the phase shifter is a supposition of a bunch of Gaussian-envelop plane waves with different random relative phases, intensity centers, and similar variances which can be expressed as

3 $${A}_{s}({\bar{z}}_{p},t)={\sum }_{j=1}^{n}{A}_{j}{e}^{-\frac{{(t-{t}_{j})}^{2}}{4{\sigma }_{t}^{2}}}{e}^{i(\omega t+{\phi }_{j})}$$

Therefore, the ${A}_{c}(\bar{z},t)$ can be further expressed as,

4 $$\begin{array}{ccc}{A}_{c}(\bar{z},t) & = & {\sum }_{j=1}^{n}{A}_{j}[{e}^{-\frac{{(t-{t}_{j})}^{2}}{4{\sigma }_{t}^{2}}}{e}^{i(\omega t+{\phi }_{j})}+\\  &  & {e}^{-\frac{{(t+\Delta t-{t}_{j})}^{2}}{4{\sigma }_{t}^{2}}}{e}^{i[\omega (t+\Delta t)+{\phi }_{j}+\frac{\pi }{6}]}(\bar{z}-{\bar{z}}_{p})]\end{array}$$

Equation (4) shows that basically the radiation field is formed by *n* modes. Each mode contains two Gaussian-envelop waves, in which the first one comes from the SASE laser pulse generated in the first undulator system and the second one comes from the microbunched electron beam coherent emission. Also, we can see that the after-delay radiator length $\bar{z}-{\bar{z}}_{p}$ determines the second wave amplitude, so that to maximize the contrast, we should set the $\bar{z}-{\bar{z}}_{p}=1$, which means the after-delay radiator length should be one gain length. Then, the x-ray pulse energy can be expressed as,

5 $$\begin{array}{ccl}W & = & \int | {A}_{c}(\bar{z},t){| }^{2}dt\\  & = & 2\sqrt{2\pi }{\sigma }_{t}{\sum }_{j=1}^{n}{A}_{j}^{2}\left[1+{e}^{-\frac{\Delta {t}^{2}}{8{\sigma }_{t}^{2}}}cos\left({\omega }_{r}\Delta t+\frac{\pi }{6}\right)\right]\end{array}$$

in which quite a lot of terms counteract each other due to the sum of independent random phases. It is clear that, the x-ray pulse energy is a combination of pulse energy generated in the before-delay part, the microbunched electron beam coherent emission and their interference. The pulse energy would oscillate with a frequency of *ω*_*r*_ = 2*πc*∕*λ* and the oscillation amplitude is a Gaussian function with $4{\sigma }_{t}^{2}$ as its variance. Here, we can regard the exponential term as a slowly varying amplitude of the oscillation. To extract the information of the coherence time, we calculate the variance of the pulse energy which can be obtained by,

6 $$\begin{array}{ccc}\bar{{(W-\bar{W})}^{2}} & = & \frac{\omega }{2m\pi }{\int }_{{t}_{0}}^{{t}_{0}+\frac{2m\pi }{\omega }}{(W-\bar{W})}^{2}dt\\  & = & 4\pi {\sigma }_{t}^{2}{({\sum }_{j=1}^{n}{A}_{j}^{2})}^{2}{e}^{-\frac{\Delta {t}^{2}}{4{\sigma }_{t}^{2}}}\end{array}$$

where *m* is a small integer. Hence, by calculating the variance of the pulse energy in several wavelengths, we can obtain the value of *σ*_*t*_ by Gaussian fitting. According to the definition of coherence time by first-order time correlation function,

7 $$\begin{array}{ccc}{\tau }_{c} & = & {\int }_{-\infty }^{\infty }| \frac{ < {A}_{c}(t){A}_{c}^{* }(t+\tau ) > }{\sqrt{ < | {A}_{c}(t){| }^{2} >  < | {A}_{c}(t+\tau ){| }^{2} > }}{| }^{2}d\tau \\  & = & \sqrt{4\pi }{\sigma }_{t}\end{array}$$

the coherence time of the x-ray pulse can be resolved.

## Supplementary information

LaTeX Supplementary File.

LaTeX Supplementary File.

LaTeX Supplementary File.

## Data Availability

The experimental data and computer code used in this paper are available from the corresponding authors upon reasonable request.

## References

[1] Emma P (2010). First lasing and operation of an Ångstrom wavelength free-electron laser. Nature Photonics.

[2] Ishikawa T (2012). A compact x-ray free-electron laser emitting in the sub-Ångström region. Nature Photonics.

[3] Kang H-S (2017). Hard x-ray free-electron laser with femtosecond-scale timing jitter. Nature Photonics.

[4] Altarelli M (2011). The european x-ray free-electron laser facility in hamburg. Nuclear Instruments and Methods in Physics Research Section B: Beam Interactions with Materials and Atoms.

[5] Milne CJ (2017). Swissfel: The swiss x-ray free electron laser. Applied Sciences.

[6] Zhang W (2014). Tracking excited-state charge and spin dynamics in iron coordination complexes. Nature.

[7] Seibert MM (2011). Single mimivirus particles intercepted and imaged with an x-ray laser. Nature.

[8] Milathianaki D (2013). Femtosecond visualization of lattice dynamics in shock-compressed matter. Science.

[9] Hartmann N (2018). Attosecond time-energy structure of x-ray free-electron laser pulses. Nature Photonics.

[10] Young L (2010). Femtosecond electronic response of atoms to ultra-intense x-rays. Nature.

[11] Eisebitt S (2004). Lensless imaging of magnetic nanostructures by x-ray spectro-holography. Nature.

[12] Marcus G, Penn G, Zholents AA (2014). Free-electron laser design for four-wave mixing experiments with soft-x-ray pulses. Phys. Rev. Lett..

[13] Roling S (2011). Temporal and spatial coherence properties of free-electron-laser pulses in the extreme ultraviolet regime. Phys. Rev. ST Accel. Beams.

[14] Mitzner R (2008). Spatio-temporal coherence of free electron laser pulses in the soft x-ray regime. Opt. Express.

[15] Osaka T (2017). Characterization of temporal coherence of hard X-ray free-electron laser pulses with single-shot interferograms. IUCrJ.

[16] Geloni, G., Kocharyan, V. and Saldin, E. Ultrafast x-ray pulse measurement method. Report No. DESY 10-008 (2010).

[17] Ding Y (2012). Femtosecond x-ray pulse characterization in free-electron lasers using a cross-correlation technique. Phys. Rev. Lett..

[18] Bonifacio R, De Salvo L, Pierini P, Piovella N, Pellegrini C (1994). Spectrum, temporal structure, and fluctuations in a high-gain free-electron laser starting from noise. Phys. Rev. Lett..

[19] Wu, J. *et al*. X-ray Spectra and Peak Power Control with iSASE. In Proceedings, 4th International Particle Accelerator Conference (IPAC 2013): Shanghai, China, p. 2068, http://JACoW.org/IPAC2013/papers/weodb101.pdf (2013).

[20] McNeil BWJ, Thompson NR, Dunning DJ (2013). Transform-limited x-ray pulse generation from a highbrightness self-amplified spontaneous-emission free-electron laser. Phys. Rev. Lett..

[21] Xiang D, Ding Y, Huang Z, Deng H (2013). Purified self-amplified spontaneous emission free-electron lasers with slippage-boosted filtering. Phys. Rev. ST Accel. Beams.

[22] Bonifacio R, Pellegrini C, Narducci L (1984). Collective instabilities and high-gain regime in a free electron laser. Optics Communications.

[23] Andruszkow J (2000). First observation of self-amplified spontaneous emission in a free-electron laser at 109 nm wavelength. Phys. Rev. Lett..

[24] Wu, J. *et al*. Lcls x-ray pulse duration measurement using the statistical fluctuation method. In Proceedings, 32nd International Free Electron Laser Conference (FEL 2010): Malmö, Sweden, p. 147, http://accelconf.web.cern.ch/AccelConf/FEL2010/papers/mopc14.pdf (2010).

[25] Bostedt C (2016). Linac coherent light source: The first five years. Rev. Mod. Phys..

[26] Feng Y (2011). A single-shot intensity-position monitor for hard x-ray fel sources. Proc. SPIE.

[27] Saldin, E. L., Schneidmiller, E. A. and Yurkov, M. V.*The physics of free electron lasers* (Springer, 2011).

[28] Reiche S (1999). Genesis 1.3: a fully 3d time-dependent FEL simulation code. Nuclear Instruments and Methods in Physics Research Section A: Accelerators, Spectrometers. Detectors and Associated Equipment.

[29] Yu LH (2000). High-gain harmonic-generation free-electron laser. Science.

[30] Amann J (2012). Demonstration of self-seeding in a hard-x-ray free-electron laser. Nature Photonics.

[31] Wu J (2007). Interplay of the chirps and chirped pulse compression in a high-gain seeded free-electron laser. Journal of the Optical Society of America B.

[32] Emma, P. *et al*. Linear accelerator design for the LCLS-II FEL facility. In Proceedings, 36th International Free Electron Laser Conference (FEL 2014), Basel, Switzerland, p. 743, http://accelconf.web.cern.ch/AccelConf/FEL2014/papers/thp025.pdf (2014).

[33] Marinelli, A. Towards Attosecond Science at LCLS and LCLSII. In Proceedings, 9th International Particle Accelerator Conference (IPAC 2018), Vancouver, BC, Canada; http://accelconf.web.cern.ch/AccelConf/ipac2018/talks/wexgbd3_talk.pdf (2018).

